# The use of metformin for type 2 diabetes prevention: Observational multicenter study from Saudi Arabia

**DOI:** 10.3389/fpubh.2022.989072

**Published:** 2022-09-08

**Authors:** Osamah M. Alfayez, Alanoud A. Alsallum, Adnan F. Aljabri, Faisal S. Almutairi, Ola Al-Azzeh, Ohoud S. Almalki, Majed S. Al Yami, Omar A. Almohammed

**Affiliations:** ^1^Department of Pharmacy Practice, College of Pharmacy, Qassim University, Qassim, Saudi Arabia; ^2^Department of Pharmacy Practice, College of Pharmacy, King Saud bin Abdulaziz University for Health Sciences, Riyadh, Saudi Arabia; ^3^Department of Clinical Pharmacy, College of Pharmacy, Taif University, Taif, Saudi Arabia; ^4^Department of Clinical Pharmacy, College of Pharmacy, King Saud University, Riyadh, Saudi Arabia; ^5^Pharmacoeconomics Research Unit, College of Pharmacy, King Saud University, Riyadh, Saudi Arabia

**Keywords:** prediabetes, diabetes prevention, metformin, type 2 diabetes, observational study

## Abstract

**Background:**

Patients with prediabetes are at higher risk of developing type 2 diabetes. While intensive lifestyle modification is the primary approach to delaying diabetes, metformin has been shown to be effective, especially among patients younger than 60 years and obese (body mass index (BMI) > 35 kg/m^2^), patients with fasting blood glucose ≥ 6.1 mmol/L or HbA1c ≥ 6%, and women with history of gestational diabetes. Thus, metformin is now recommended as an option for diabetes prevention by the American Diabetes Association (ADA). The use of metformin among patients with prediabetes in Saudi Arabia and their adherence to the guideline's recommendation for the prevention of type 2 diabetes is unknown. This study aimed to identify the prevalence of metformin use among prediabetes patients overall and patients who are more likely to benefit from metformin use per the ADA guidelines.

**Methods:**

A retrospective cohort study was conducted encompassing data from three tertiary care hospitals between January 2015 and June 2019. All patients aged 20 to 70 years with prediabetes (HbA1c of 5.7–6.4%) were included, while patients with an established diagnosis of diabetes, creatinine clearance <45 ml/min, using antihyperglycemic medications other than metformin, or on metformin for other indications were excluded. Prediabetes patients who are most likely to benefit from metformin for type 2 diabetes prevention are those younger than 60 years with a BMI ≥ 35 kg/m^2^, patients with fasting blood glucose ≥ 6.1 mmol/L or HbA1c ≥ 6%, and women with history of gestational diabetes. This study examined the prevalence of metformin use among all patients with prediabetes, as well as patients who would be more likely to benefit from metformin use per the ADA guidelines.

**Results:**

A total of 251 patients were included in this study; 52.2% were female, with a mean age of 47.0 (11.9) years and BMI of 32.3 (6.5) kg/m^2^, and the median HbA1c at baseline was 5.8% (5.7–6.0). Among the overall sample, 18 patients (7.2%) received metformin for the prevention of type 2 diabetes, 14 of those were from the groups that are more likely to benefit from metformin use per the ADA guidelines (9.9%).

**Conclusions:**

Among individuals with prediabetes in Saudi Arabia, metformin use was very low despite the evidence supporting its safety, convenience, and efficacy. Healthcare providers seemed hesitant to medicalize prediabetes; furthermore, the low use of metformin suggests the existence of several barriers that need to be identified and resolved. Increasing providers' knowledge and awareness regarding screening and management of prediabetes is highly encouraged.

## Introduction

Patients with prediabetes are at a higher risk of developing type 2 diabetes (1). One major approach to delaying diabetes is through intensive lifestyle modifications that focus on weight loss and exercise (2, 3). The landmark trial of the diabetes prevention program (DPP) identified intensive lifestyle modification as the best intervention to delay the incidence of type 2 diabetes (2). Unfortunately, the translation of this experience outside the controlled setting of the clinical trial demonstrated inconsistent results (4, 5).

Although intensive lifestyle modification was shown to be superior to metformin in reducing the risk of diabetes (58 vs. 31%, respectively) in the DPP trial, metformin demonstrated a 53% reduction in the risk of diabetes among those who were obese, with a body mass index (BMI) of > 35 kg/m^2^, and younger than 60 years of age as well as women with a history of gestational diabetes compared to 51% with the intensive lifestyle modification (2). For metformin, the reduction in the incidence of type 2 diabetes compared to placebo was 31% in the DPP trial and 18% in 10-year and 15-year follow-up studies (2, 3). The standard of medical care in diabetes guidelines published by the American Diabetes Association (ADA) recommends the use of metformin as an option for diabetes prevention, especially among the aforementioned high-risk group (1). However, the use of metformin for this indication is off label according to the summary of product characteristics (SPC).

Despite the existing evidence, the use of lifestyle modifications and metformin for diabetes prevention remains underutilized (6–9). Mainous and colleagues found that lifestyle modifications or metformin were only initiated in 23% of patients with prediabetes (6, 7). Another study found that <5% of prediabetes patients were referred to lifestyle modification programs; furthermore, metformin was used in <1% of these patients. A related study found that among the eligible patients, only 7.8% received metformin (9). Several barriers to adopting a healthy lifestyle among people with obesity/newly diagnosed diabetes or prediabetes have been reported. For example, at the patient level, poor diet education, lack of adequate understanding of a healthy diet, lack of motivation, low socioeconomic status, emotional stress, presence of depression, and lack of symptoms have been reported to impact the initiation and maintenance of a healthy lifestyle. There are also factors at both physician and health system levels such as the limited resources, insufficient time for physicians, failure to identify patients' needs, hesitation to refer patients to other healthcare providers, limited integration of modern technologies, lack of a team-based approach, and limited community outreach (10). All those factors make it difficult for patients to adopt a healthy lifestyle.

The prevalence of prediabetes and type 2 diabetes in Saudi Arabia has been found to vary significantly in the available literature (11–13). Regardless of the true prevalence, type 2 diabetes remains a serious health issue that increases the burden on the Saudi healthcare system (14). The use of metformin among patients with prediabetes and their adherence to the clinical guidelines is unknown. Thus, in this study, we aimed to identify the prevalence of metformin use among prediabetes patients in three regions of Saudi Arabia, along with the prevalence of metformin use among patients with prediabetes who would be more likely to benefit from metformin use per the ADA guidelines.

## Methods

### Study design, setting, and participants

A retrospective cohort study was conducted, that considered data from three tertiary care hospitals in Saudi Arabia between January 2015 and December 2019. We included all patients aged 20 to 70 years with prediabetes (HbA1C of 5.7–6.4%) based on the ADA definition (15). Patients were excluded if they had an established diagnosis of diabetes, glomerular filtration rate or creatinine clearance < 45 ml/min, or were on antihyperglycemic medications other than metformin, as well as patients on metformin for other indications, such as polycystic ovary syndrome.

### Data collection and outcome measures

Data collected from the electronic health records included demographic variables, BMI, comorbid conditions, laboratory values for HbA1c, fasting and random blood glucose (FBG and RBG, respectively), vitamin B12, and lipid profile at baseline, the follow-up value for HbA1c, and the name and strength of medications used by these patients—specifically, the use of metformin and statins—as well as the documentation of developing type 2 diabetes in the patient record. Prediabetes patients who are most likely to benefit from metformin for the prevention of type 2 diabetes are those aged < 60 years with BMI ≥ 35 kg/m^2^, patients with FBG ≥ 6.1 mmol/L or HbA1c ≥ 6%, and women with history of gestational diabetes. Thus, these factors were used to assess adherence to the ADA recommendations regarding the prevention of type 2 diabetes. The endpoints included the prevalence of metformin use among prediabetes patients overall and the prevalence of metformin use among patients who were more likely to benefit from metformin per the ADA guidelines.

### Statistical analysis

Descriptive statistics, mean with standard deviation (SD) or median with interquartile range (IQR) and frequency with percentage (%) were used to describe continuous and categorical data, respectively. Patients' data were presented in tables based on the use of metformin according to the guidelines' recommendations: patients prescribed metformin and those not prescribed metformin. Data were collected and managed using Microsoft Excel, version 2010 (Microsoft Corp., Redmond, WA, USA), and all statistical analyses were conducted using the SAS® software, version 9.4 (SAS Institute Inc., Cary, NC, USA).

## Results

A total of 251 patients with prediabetes were included in this study, of which 131 (52.2%) were female. The mean age of these patients was 47.0 (11.9) years, and their BMI was 32.3 (6.5) kg/m^2^. The most recorded comorbidity was dyslipidemia (36.7%), followed by hypertension (25.5%), and statin therapy was used in 37.5% of the sample (Table 1). The median HbA_1_c at baseline was 5.8% (5.7–6.0), and the mean FBG and RBG levels were 5.5 and 5.7 mmol/L, respectively (Table 2).

**Table 1:** Patients' characteristics (*n* = 251).

**Characteristic**	**Number (%)**
Age, in years	47.0 (11.9)
Gender	
Male	120 (47.8)
Female	131 (52.2)
BMI, kg/m^2^	32.3 (6.5)
**Comorbidities**	
Dyslipidemia	92 (36.7)
Hypertension	64 (25.5)
Coronary artery disease	17 (6.8)
History of gestational diabetes	14 (5.6)
Renal disease	10 (4.0)
Heart failure	7 (2.8)
Chronic heart disease	4 (1.6)
Arrhythmia	4 (1.6)
Statins use	94 (37.5)

Data presented as number (%) or mean (SD).

**Table 2:** Patients' baseline laboratory data.

**Laboratory test**	**Mean (SD)**
Baseline HbA1c, %	5.9 (0.2)
Baseline HbA1c, %	5.8 (5.7–6.0)
FBG, mmol/L	5.5 (0.7)
RBG, mmol/L	5.7 (1.1)
Baseline vitamin B12, pmol/L	238 (172–348)
**Baseline lipid profile**	
LDL, mmol/L	3.1 (0.9)
HDL, mmol/L	1.2 (0.4)
Triglyceride, mmol/L	1.5 (1.2)

Data presented as mean (SD) or median (IQR).

Hb A1c, hemoglobin A1c; SD, Standard Deviation; IQR, Interquartile range; FBG, Fasting blood glucose; RBG, Random blood glucose; HDL, High-density lipoprotein; LDL, Low-density lipoprotein.

Among the entire sample of prediabetes patients, 18 (7.2%) received metformin for the prevention of type 2 diabetes (Table 3). Among those who received metformin, the most common comorbidities were dyslipidemia (55.6%), followed by hypertension (33.3%). The mean HbA_1_c, FBG, and RBG at baseline were 6.0%, 5.8 mmol/L, and 6.0 mmol/L, respectively, in those who received metformin, compared with 5.9%, 5.4 mmol/L, and 5.6 mmol/L in those who did not receive metformin (Table 4). The initial doses of metformin were 500 mg once daily in 9 patients (50%) and 500 mg twice a day in 7 patients (38.9%). Statins were used by 55.6% of these patients, compared to 36.1% of those who were not on metformin.

**Table 3:** Patients' characteristics based on the prescribing of metformin.

**Characteristics**	**Prescribed**	**Not prescribed**
	**metformin**	**metformin**
	***n* = 18 (7.2)**	***N* = 233 (92.8)**
Age, years	50.2 (11.8)	46.8 (11.9)
**Gender**		
Male	10 (55.6)	110 (47.2)
Female	8 (44.4)	123 (52.8)
BMI, kg/m^2^	33.2 (4.9)	32.3 (6.6)
BMI ≥ 35 kg/m^2^	5 (27.7)	52 (91.2)
**Comorbidities**		
Hypertension	6 (33.3)	58 (24.9)
Dyslipidemia	10 (55.6)	82 (35.2)
Chronic heart disease	0 ()	4 (1.7)
Coronary artery disease	2 (11.1)	15 (6.4)
Arrhythmia	0 (0)	4 (1.7)
Heart failure	0 (0)	7 (3.0)
Renal disease	0 (0)	10 (4.3)
History of gestational diabetes	1 (5.6)	13 (5.6)
Statin use	10 (55.6)	84 (36.1)
**Initial metformin doses prescribed**		
500 mg QD	9 (50.0)	—
500 mg BID	7 (38.9)	—
500 mg TID	1 (5.6)	—
1000 mg TID	1 (5.6)	—

Data presented as number (%) or mean (SD).

BMI, Body Mass Index; SD, Standard Deviation; IQR, Interquartile range; QD, One time daily; BID, Two times daily; TID, Three times daily.

**Table 4:** Patients' baseline laboratory data based on the prescribing of metformin.

**Laboratory tests**	**Prescribed**	**Not prescribed**
	**metformin**	**metformin**
	***n* = 18 (7.2)**	***n* = 233 (92.8)**
Baseline HbA1c, %	6.0 (0.2)	5.9 (0.2)
fFBG, mmol/L	5.8 (0.6)	5.4 (0.7)
RBG, mmol/L	6.0 (1.5)	5.6 (1.1)
Baseline vitamin B12, pmol/L	168 (148–215)	242 (174–360)
**Lipid profile**		
LDL, mmol/L	2.8 (0.9)	3.1 (0.9)
HDL, mmol/L	1.1 (0.3)	1.2 (0.4)
Triglyceride, mmol/L	2.2 (2.8)	1.4 (0.9)

Data presented as mean (SD) or median (IQR).

Hb A1c, hemoglobin A1c; SD, Standard Deviation; IQR, Interquartile range; FBG, Fasting blood glucose; RBG, Random blood glucose; HDL, High-density lipoprotein; LDL, Low-density lipoprotein.

**Figure 1:**
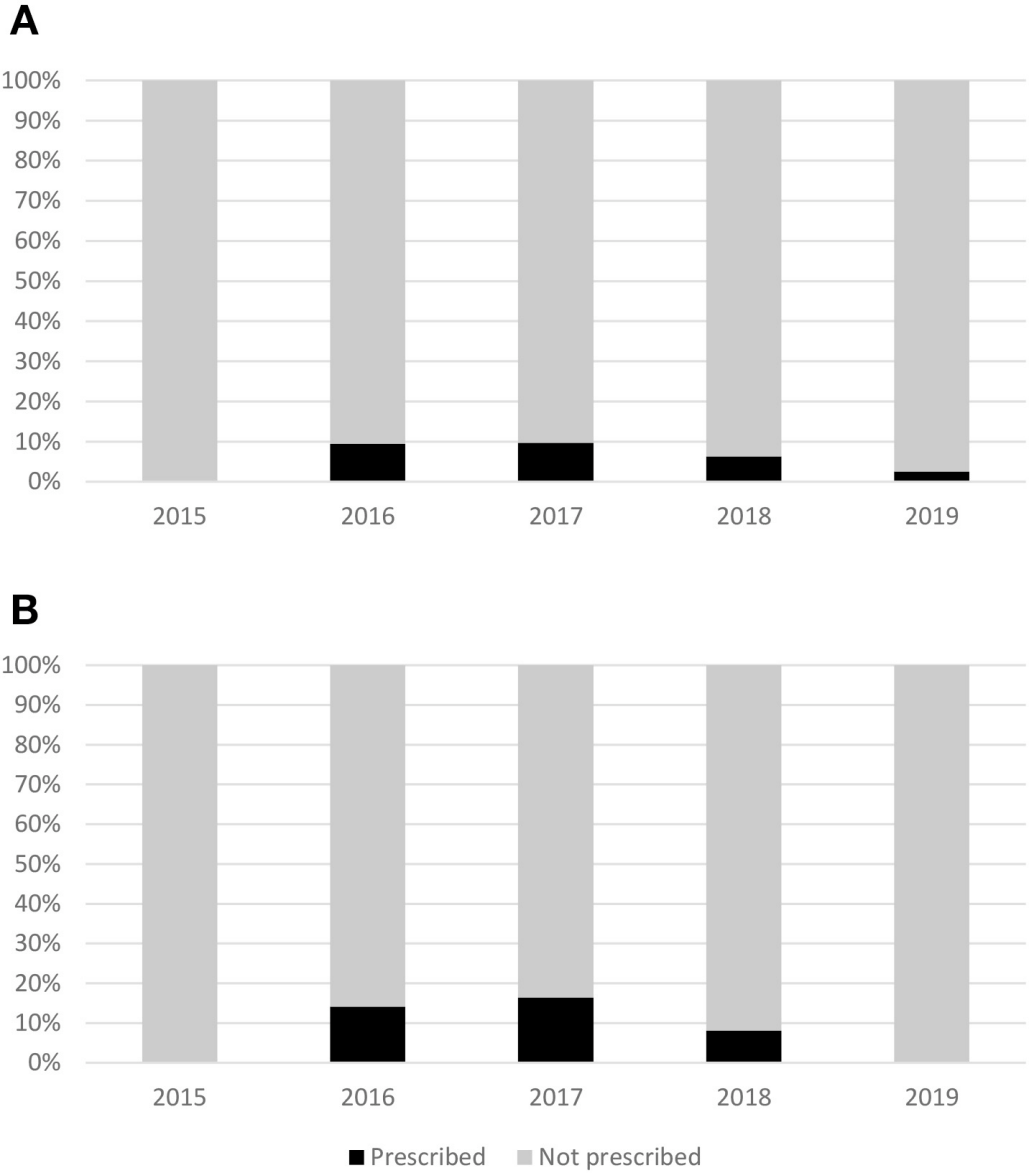
Trend of prescribing metformin for diabetes prevention for the overall population **(A)** and patients that are more likely to benefit from metformin **(B)**.

Regarding the group that was more likely to benefit from metformin and thus encouraged to receive metformin per the ADA recommendations, only 14 out of 142 patients (9.9%) received prescriptions for metformin. Among these, 17.5% of patients with FBG ≥ 6.1 mmol/L were prescribed metformin. In addition, only one out of 14 women with gestational diabetes were prescribed metformin (Table 5). Moreover, no clear trend toward the prescribing of metformin for diabetes prevention was observed during the study (Figure 1).

**Table 5:** Sub-analysis for patients who are more likely to benefit from metformin (*n* = 142).

**Characteristics**	**Prescribed**	**Not prescribed**
	**metformin**	**metformin**
Patients	14 (9.9)	128 (90.1)
**Categories**		
Age <60 years and BMI ≥ 35 Kg/m^2^	5 (8.5)	54 (91.5)
FBG ≥ 6.1 mmol/L (110 mg/dl)	7 (17.5)	33 (82.5)
HbA1c ≥ 6 %	6 (7.5)	73 (92.4)
History of gestational diabetes	1 (7.1)	13 (92.9)

Data presented as number (%).

BMI, body mass index; Hb A1c, hemoglobin A1c; FBG, Fasting blood glucose.

## Discussion

Although type 2 diabetes remains a serious health issue that increases the burden on the Saudi healthcare system, the use of metformin among patients with prediabetes is unknown. This study was conducted to identify the prevalence of metformin use among an overall sample of prediabetes patients and patients who were more likely to benefit from metformin use per the ADA guidelines. The prevalence of metformin use was only 7.2% in the overall sample and 9.9% among the subgroup of high-risk patients who were more likely to benefit from receiving metformin, based on the current evidence and the ADA guidelines' recommendations. Our study, which included individuals with prediabetes from three regions in Saudi Arabia, found metformin to be underused.

The observed underutilization of metformin for the prevention of type 2 diabetes is similar to the findings of two previous observational studies (8, 9). Schmittdiel and colleagues found that metformin was initiated in <1% of identified patients with prediabetes. Notably, the study included patients with confirmed prediabetes diagnoses between 2006 and 2010 (8). The ADA guidelines first began to recommend metformin as an option for diabetes prevention in 2008 (16). This timing might explain the extremely low rate of metformin use along with the use of data from a single electronic health record. Meanwhile, Moin et al. reported that 3.7% of working, insured adults with prediabetes received metformin. Moreover, in the subcategory of patients with a BMI of more than 35 kg/m^2^ or who had a history of gestational diabetes, 7.8% of patients received metformin. That study used a nationwide database for a large insurer in the United States that included data for working employees between 2010 and 2012 (9).

Research has shown that metformin is safe, convenient, and cost-effective when used for the prevention of type 2 diabetes (17, 18). For example, one metanalysis revealed the absolute risk reduction observed with metformin use ranged from 4.4 to 14.3% (17). From the population perspective, such numbers are critical in the case of a disease with a high prevalence, such as type 2 diabetes. Moreover, in a recent systematic review by Moin et al. the researchers suggested that metformin might be associated with higher absolute risk reduction, especially among certain higher-risk patients, such as those who were morbidly obese, as well as patients with higher HbA1c or Impaired FBG values (19). The difficulty in implementing lifestyle modification-based programs into real practice increases the importance of using metformin for diabetes prevention, at least among high-risk patients who are more likely to benefit from the use of metformin. Unfortunately, the low use of metformin indicates the existence of several barriers to implementing this approach.

As suggested by Moin et al. the lack of eligibility criteria as to who should receive metformin might have been an important challenge (19), especially since patients with prediabetes vary in their risk for developing type 2 diabetes. Health care providers' knowledge regarding prediabetes screening and treatment might be another barrier. Tseng et al. surveyed primary care providers to assess their knowledge of screening and managing prediabetes. The study findings revealed that only 6% managed to identify the correct risk factors to warrant prediabetes screening; in addition, only 11% of the primary health care providers mentioned that they would refer patients with prediabetes to a weight-loss program. moreover, only 17% managed to identify the correct lab values for diagnosing prediabetes (20). This study highlights a critical barrier that needs to be addressed, especially in primary care settings.

In Saudi Arabia, a study has been carried out to assess physicians' knowledge and attitudes regarding screening, diagnosing, or managing prediabetes (21). In that study, 115 primary care physicians (pcps) participated in a national survey. the study authors reported that only 27% of the study participants were aware of the risk factors for prediabetes. in addition, only 55.5 and 43.6% of the participants were aware of the HbA1c and FBG levels, respectively, for the diagnosis of prediabetes. Around 11.0% of the participating PCPs reported the use of metformin in more than half of their prediabetes patients. However, the participants were not asked about reasons for not starting metformin; that said, it is possible that the lack of an FDA indication for diabetes prevention on the metformin label and the possible side effects that might be associated with metformin use might have affected health care providers' attitudes toward prescribing metformin.

In order to overcome the previously mentioned barriers, it is highly recommended to educate health care providers about the screening and management of prediabetes. Increasing health care providers' knowledge regarding risk factors, diagnostic criteria, and the clinical guidelines' recommendations will promise great benefit, especially in primary care settings. Furthermore, healthcare institutions should develop clear criteria on who should be screened for diabetes; institutions should also have a clear eligibility criterion in place regarding who should receive metformin along with lifestyle modifications. Developing a simplified protocol for the use of primary care providers that includes metformin dosage instructions, monitoring, and patient education might help encourage physicians to medicalize prediabetes. In addition, patient-related factors that might hinder managing prediabetes can be overcome by increasing patients' awareness of diabetes prevention, and the benefits of lifestyle modification, and metformin might aid in these patients' acceptance of such interventions for prevention. Physicians' lack of time and limited staffing resources might be overcome by including clinical pharmacists as part of the multidisciplinary team in ambulatory care settings, which can play a vital role in the initiation and monitoring of metformin.

Although metformin is considered a very safe medication, it has some side effects, such as gastrointestinal disorders and B12 deficiency, that may hinder the prescribing or use of this medication as a protective measure (22). In addition, lactic acidosis is a rare side effect but it could occur with the chronic use of metformin, especially in patients with renal impairment (22). While the use of metformin can reduce the incidence of type 2 diabetes, it is still unclear whether the early introduction of metformin in patients with prediabetes may affect some of the patient outcomes in later stages, such as cardiovascular diseases or mortality (22). Thus, the lack of evidence about metformin's benefits on those outcomes might hinder its initiation by some healthcare providers (22). Therefore, the results of the ongoing VA-IMPACT study, investigating the potential impact of metformin vs. placebo among people with prediabetes on atherosclerotic cardiovascular disease and mortality might provide insight into the issue (23). Besides that, there is emerging evidence that new antidiabetic agents (SGLT2i and GLP-1 RA) may have favorable effects on the risk of progression of prediabetes to diabetes (24, 25). An exploratory analysis from DAPA-HF showed that the use of dapagliflozin in patients with heart failure reduces the incidence of new diabetes by 32% (24). Furthermore, in the SCALE obesity and prediabetes trial, the use of liraglutide among patients with prediabetes and obesity showed a reduction in the risk of diabetes during follow-up (25).

The overall small sample size included in this study is a limitation that must be noted. This aspect of the study might be a result of the low screening rate for diabetes overall, especially among the youthful segment of the population. The retrospective design of this study also imposes some limitations that must not be overlooked. The results of this study should be interpreted with caution, as only three regions of the country were included; thus, the findings from the study may not be generalized to other regions in Saudi Arabia. Also, larger study that include more regions are needed to assess the situation at the country level. Data regarding lifestyle modifications and referral to lifestyle modification-based programs were not reported in the database; thus, it was not clear whether patients were given these interventions in addition to metformin. Although this investigation represents the first multicenter study to address the rate of metformin use for diabetes prevention in Saudi Arabia, additional and larger studies are needed to investigate the reasons behind the low level of adherence to this guideline-based recommendation.

## Conclusion

Among Saudi individuals with prediabetes, metformin use was very low despite the evidence supporting its safety, convenience, and efficacy. Health care providers seem to be hesitant to medicalize prediabetes, and the low use of metformin suggests the existence of possible barriers. increasing health care providers' knowledge and awareness regarding the screening and management of prediabetes is highly encouraged. The Ministry of Health, along with other healthcare settings and policymakers in saudi arabia, can utilize the results of this study to further promote the incorporation of evidence-based diabetes preventive measures into real-world practice, including referral of all patients to life style modification program and the use of metformin for patients who are more likely to benefit from it according to the ADA recommendations.

## Data availability statement

The original contributions presented in the study are included in the article/supplementary material, further inquiries can be directed to the corresponding author/s.

## Ethics statement

The studies involving human participants were reviewed and approved by Institutional Review Board at King Abdullah International Medical Research Center. Written informed consent for participation was not required for this study in accordance with the national legislation and the institutional requirements.

## Author contributions

OMA, MA, and OAA contributed to the conception and design of this study. OAA conducted the statistical analysis and wrote the section Methods with inputs from OMA, MA, and OSA. AAA, AFA, FA, and OA-A did the data collection and wrote the section Results. OMA is the guarantor of this work. All authors contributed to writing the manuscript and revised and approved the final version of the manuscript.

## Funding

This research was funded by the Researcher Supporting Project (RSP-2021/77), King Saud University, Riyadh, Saudi Arabia.

## Conflict of interest

The authors declare that the research was conducted in the absence of any commercial or financial relationships that could be construed as a potential conflict of interest.

## Publisher's note

All claims expressed in this article are solely those of the authors and do not necessarily represent those of their affiliated organizations, or those of the publisher, the editors and the reviewers. Any product that may be evaluated in this article, or claim that may be made by its manufacturer, is not guaranteed or endorsed by the publisher.
